# Domestication via the commensal pathway in a fish-invertebrate mutualism

**DOI:** 10.1038/s41467-020-19958-5

**Published:** 2020-12-07

**Authors:** Rohan M. Brooker, Jordan M. Casey, Zara-Louise Cowan, Tiffany L. Sih, Danielle L. Dixson, Andrea Manica, William E. Feeney

**Affiliations:** 1 Centre for Integrative Ecology, School of Life and Environmental Sciences, Deakin University, Queenscliff, VIC 3225 Australia; 2 Department of Biology, University of Saskatchewan, Saskatoon, SK S7N 5A2 Canada; 3 School of Marine Science and Policy, University of Delaware, Lewes, DE 19958 USA; 4 PSL Université Paris: EPHE-UPVD-CNRS, USR 3278 CRIOBE, Université de Perpignan, 66000 Perpignan, France; 5 Laboratoire d’Excellence “CORAIL”, Perpignan, France; 6 Department of Invertebrate Zoology, National Museum of Natural History, Smithsonian Institution, Washington, DC 20560 USA; 7 Environmental Futures Research Institute, Griffith University, Nathan, QLD 4222 Australia; 8 Department of Zoology, University of Cambridge, Cambridge, CB2 1TN UK; 9 School of Biological Sciences, Monash University, Melbourne, VIC 3800 Australia; 10 Department of Behavioural Ecology and Evolutionary Genetics, Max Planck Institute for Ornithology, 82319 Seewiesen, Germany; 11 School of Biological Sciences, University of Queensland, Brisbane, QLD 4072 Australia

**Keywords:** Behavioural ecology, Community ecology, Evolutionary ecology, Marine biology

## Abstract

Domesticator-domesticate relationships are specialized mutualisms where one species provides multigenerational support to another in exchange for a resource or service, and through which both partners gain an advantage over individuals outside the relationship. While this ecological innovation has profoundly reshaped the world’s landscapes and biodiversity, the ecological circumstances that facilitate domestication remain uncertain. Here, we show that longfin damselfish (*Stegastes diencaeus*) aggressively defend algae farms on which they feed, and this protective refuge selects a domesticator-domesticate relationship with planktonic mysid shrimps (*Mysidium integrum*). Mysids passively excrete nutrients onto farms, which is associated with enriched algal composition, and damselfish that host mysids exhibit better body condition compared to those without. Our results suggest that the refuge damselfish create as a byproduct of algal tending and the mutual habituation that damselfish and mysids exhibit towards one another were instrumental in subsequent mysid domestication. These results are consistent with domestication via the commensal pathway, by which many common examples of animal domestication are hypothesized to have evolved.

## Abstract (web summary)

It has been hypothesized that domestication can occur through the ‘commensal pathway’ in which the domesticate takes advantage of a niche created as a byproduct by the domesticator. Here, Brooker et al. provide evidence for a commensal domestication process between longfin damselfish and mysid shrimps.

## Introduction

Domesticator-domesticate relationships are ecologically important interactions that have shaped the world’s landscapes and biodiversity^1,2^. While the processes associated with the evolution of these relationships remain a subject of ongoing interest^3–6^, our understanding has progressed markedly since Darwin first documented the convergence of characteristics diagnostic of domesticated species (i.e., a ‘domestication syndrome’) over 150 years ago^7^. Initially, the presence of behavioral and morphological traits in domesticated species that directly support the relationship but have limited apparent value outside of that purpose led to the interpretation that domestication was always the product of human intent^8^. This notion was challenged by evidence demonstrating that these traits could arise without human-imposed selection^9,10^, and the identification of domesticator-domesticate relationships between non-human species^11–15^. With these relationships broadly defined as a specialized form of mutualism where one species provides prolonged support to another in exchange for a predictable resource or service^5^, it is hypothesized that the domestication of animals can occur via one of three pathways^4,16,17^: (i) a ‘commensal’ pathway, (ii) a ‘prey’ pathway, and (iii) a ‘directed’ pathway. While the prey and directed pathways require intentional behaviors on the part of the proto-domesticator that have only been observed in humans, the commensal pathway posits that domestication can emerge when an opportunistic species utilizes a niche created as a byproduct of another species’ behavior^5^. This pathway is suspected to be that by which relationships between humans and many familiar animals such as dogs, cats, chickens and pigs^4^ emerged.

Evidence of the behavioral processes that support the emergence of animal domestication remain surprisingly elusive. It is generally considered that domestication via the commensal pathway is underpinned by: (i) a proto-domesticate being attracted to a niche created by a proto-domesticator, (ii) both species becoming habituated towards one another’s presence, and (iii) the one-way commensalism progressing into a mutually beneficial relationship^4,16,17^. Following these foundational steps, positive feedback loops can enable the further progression towards more specialized behaviors, such as dominion over reproduction, and morphological and genetic differentiation from wild types. For example, regarding the domestication of gray wolves (*Canis lupus*) by humans, it is hypothesized that wolves were initially attracted to human encampments, that humans and wolves progressively became more habituated towards one another, and that humans eventually derived benefits through their relationships with wolves such as increased hunting proficiency^9,18^. While the domestication of an animal by a non-human species has yet to be identified, non-human domesticator-domesticate relationships do exist, exemplified by the various insects (ants, beetles, and termites) that have domesticated fungi^11–13^. While the pathways to fungal domestication differ from those proposed for animals, the ability of the proto-domesticate to occupy a novel niche may have also played a role in facilitating these relationships. For instance, phylogenetic analyses of fungus-growing ants suggests that while non-obligate fungal tending first emerged in tropical rainforests^13,19^, obligate domestication subsequently evolved in dry habitats inhospitable to free-living fungi^20^. Under these conditions, the ability of fungi to persist within nests increased its reliance on ants and facilitated its domestication. However, while archeological and evolutionary evidence for the role of novel niche use and for the commensal pathway exists^20,21^, experimental support for the foundational behavioral steps has been difficult to obtain due to the typically obligate nature of domesticator-domesticate relationships.

In this work, we show that interactions between algae-farming damselfishes (Pomacentridae) and farm-associated mysid shrimps (Mysidae) constitute a domesticator-domesticate relationship, and that this relationship can provide new insights into the process of animal domestication. Numerous coral reef-associated damselfishes engage in territorial farming behaviors^22^, which are characterized by the tending and defense of turf-algae patches (herein ‘farms’) on which they primarily feed^23^. Reliance on algae varies across species, spanning from facultative to obligate, with highly dependent species aggressively defending their smaller, more specialized farms^24^. Filter-feeding mysids are common components of Caribbean reef communities, serving as an important prey item for many fishes^25^. Despite the predatory risk posed by farming damselfishes^26^, mysid swarms have been reported to aggregate within the territories of several damselfishes in the Caribbean^27–29^ and exhibit site fidelity over an extended period^29^; however, the nature of these relationships remains unknown. As metabolic excretions can provide supplementary nutrition to marine plants and sessile organisms^30^, mysid waste may enrich the associated algal community, providing mysid-associated fishes with higher quality food. If the relationship between damselfish and mysids is mutually beneficial, it may be actively maintained and constitute domestication via natural selection.

## Results

### Associations between mysids and farming damselfishes

**Fig. 1:**
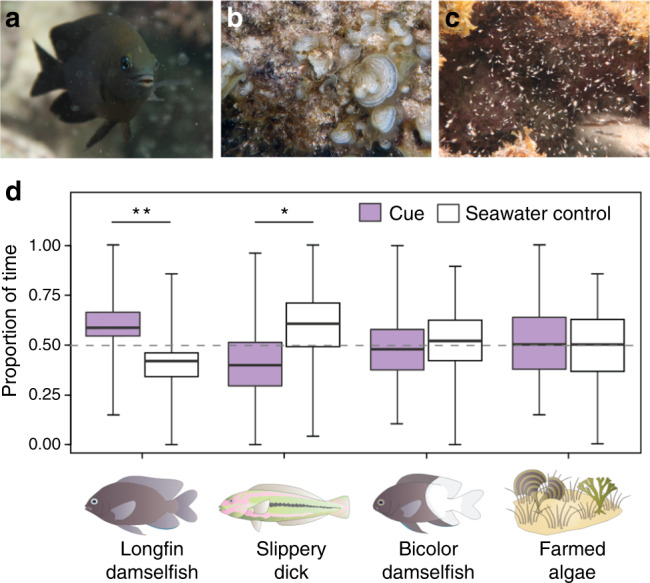
Study system and mysid olfactory preferences. (**a**) The longfin damselfish, *Stegastes diencaeus*, (**b**) detail of algal ‘farm’, comprised of both turf-algae and brown algae (Ochrophyta), including *Dictyota* and *Padina*, (**c**) swarm of mysid shrimps, *Mysidium integrum*. (**d**) Proportion of time mysids spent in water sources containing either an olfactory cue (purple) or blank seawater (white) during two-channel choice flume experiments. Cues were (left to right), the proposed domesticator (longfin damselfish, *S. diencaeus*) (Wilcoxon signed-rank test: *V* = 358, *P* = 0.002, *n* = 30 trials), a mysid predator (slippery dick, *Halichoeres bivittatus)* (Paired *t*-test: *t*_*29*_ = −2.2, *P* = 0.033, *n* = 30 trials), a related non-mysid-associated damselfish (bicolor damselfish, *Stegastes partitus*) (Paired *t*-test: *t*_29_ = −0.4, *P* = 0.727, *n* = 30 trials), and farmed algae (Paired *t*-test: *t*_29_ = 0.3, *P* = 0.784, *n* = 30 trials). A different mysid shrimp individual was used in each trial. Asterisks indicate significant differences (*P* < 0.05 = *, *P* < 0.01 = **, *P* < 0.001 = ***). Boxplots show median values (horizontal lines), interquartile range (boxes), and minimum and maximum values (whiskers).

To investigate this hypothesis, we examined the three-way relationship between longfin damselfish (*Stegastes diencaeus*) (Fig. 1a), the algae community they intensively maintain (Fig. 1b), and the planktonic mysids (*Mysidium integrum*) (herein ‘mysids’; Fig. 1c) that associate with damselfish farms around Carrie Bow Cay, Belize (16°48.15′N, 88°04.95′W). To examine association patterns between species, we first conducted transects along the reef flat. While not all farms contained mysid swarms, mysids were always associated with a *Stegastes* damselfish. Mysids were significantly more likely to associate with intensive-farming species, such as the longfin damselfish (31.17%, *n* = 143) or its ecologically similar sister species^31^, the dusky damselfish (*Stegastes adustus*) (13.33%, *n* = 45) (but not with threespot damselfish [*S. planifrons*] [0%, *n* = 7]), compared to sympatric, non-intensive-farming species, such as the cocoa damselfish (*S. variabilis*) (10%, *n* = 10), beaugregory (*S. leucostictus*) (4.91%, *n* = 81), or bicolor damselfish (*S. partitus*) (2.13%, *n* = 47) (26.67% of intensive-farming fishes [*n* = 195] versus 4.35% of non-intensive-farming fishes [*n* = 138]; $\chi _1^2$ = 29.87, *P* < 0.001). As the aim of this study was to understand the nature of the relationship between algal-farming damselfish and mysids all subsequent experiments focused on the longfin damselfish (herein ‘damselfish’) as mysids were most commonly found in association with this species.

### Mysid site fidelity and responses to habitat-related cues

Mysids from the genus *Mysidium* exhibit continuous reproduction, rapid development of internally held larvae and homing behavior^25,29,32^, suggesting prolonged associations between swarms and farms are intergenerational. Periodic censuses (day 0, 10, and 20) during the day and night of farms that either hosted (*n* = 30) or lacked (*n* = 30) mysid swarms found that mysids either consistently ($\chi _2^2$ = 2, *P* = 0.368) or never resided within farms during the day and that mysids were never within farms at night, which is consistent with both predicted diel migration patterns and intergenerational mysid-farm associations. To test how mysids re-locate to farms, we conducted olfactory choice experiments^33^ and found that mysids were attracted to longfin damselfish odor (*V* = 358, *n* = 30, *P* = 0.002), avoided diurnal predator odor (slippery dick wrasse, *Halichoeres bivittatus*) (*t*_*29*_ = −2.2, *P* = 0.033) and exhibited no response to the odor of an abundant, but less territorial damselfish (*S. partitus*) (*t*_29_ = −0.4, *P* = 0.727) (Fig. 1d). No response was shown to farmed algae odor (*t*_29_ = 0.3, *P* = 0.784), suggesting mysids are attracted to the farming damselfish rather than the farm itself (Fig. 1d).

### Effect of predation on the damselfish-mysid relationship

**Fig. 2:**
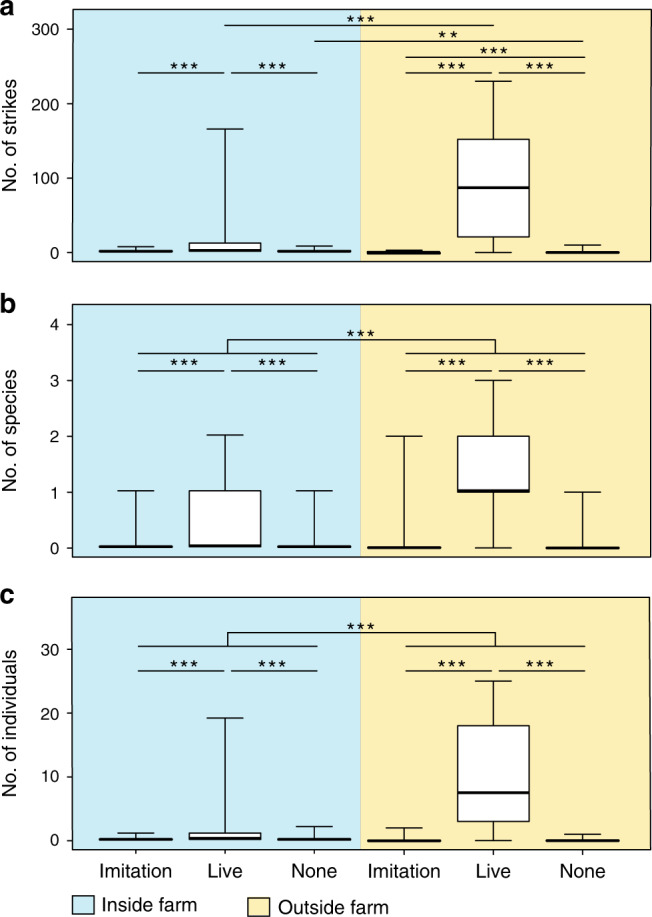
Predation risk to mysids inside and outside of longfin damselfish farms. Comparative responses by predatory fishes towards clear plastic bags containing either 150 ‘imitation’ mysid shrimps, 150 live mysid shrimps, or an empty seawater control, when placed inside (blue) versus outside (yellow) of longfin damselfish (*Stegastes diencaeus*) farms. Responses are: (**a**) number of strikes by predatory fishes on each bag (significant interaction between location and treatment (zero-inflated GLMM): $\chi _2^2$ = 7.2, *P* = 0.028), (**b**) number of predatory fish species that struck at each bag (significant effect of location (GLMM): $\chi _1^2$ = 17.6, *P* < 0.001; significant effect of treatment (GLMM): $\chi _2^2$ = 81, *P* < 0.001) and (**c**) number of individual predators that struck at each bag (significant effect of location (negative binomial GLMM): $\chi _1^2$ = 51.01, *P* < 0.001; significant effect of treatment (negative binomial GLMM): $\chi _2^2$ = 253.71, *P* < 0.001). Experiments were conducted at 30 damselfish farms, with *n* = 30 trials inside the farm and *n* = 30 trials conducted outside of the farm. Asterisks indicate significant differences (*P* < 0.05 = *, *P* < 0.01 = **, *P* < 0.001 = ***) and all posthoc tests included mvt corrections. Boxplots show median values (horizontal lines), interquartile range (boxes), and minimum and maximum values (whiskers).

To test whether associations with damselfish farms conferred protective benefits to mysids, we conducted two predation experiments. In the first experiment, responses by naturally occurring predatory fishes were recorded towards a clear plastic bag filled with: (1) 150 mysids, (2) 150 mysid-sized silicone pieces (‘imitation’ swarm), and (3) seawater. Trials were conducted within and immediately outside damselfish farms. We found a significant interaction between location and treatment on the number of predator strikes at live swarms ($\chi ^2_2$ = 7.2, *P* = 0.028; see Table S1 for model coefficients), with most variation explained by mysids experiencing more attacks compared to the control treatments, and more attacks outside of farms compared to inside of farms (Fig. 2a). We also found significant effects of location ($\chi _1^2$ = 17.6, *P* < 0.001) and treatment ($\chi _2^2$ = 81, *P* < 0.001) on the diversity of predators that attacked swarms (Fig. 2b). Significantly more species attacked live swarms compared to the empty bag control (coefficient: 3.3, *z* = 4.6, *P* < 0.001), there was no difference in the diversity of predators that attacked the control treatments (coefficient: 1.3, *z* = 1.6, *P* = 0.117) and more species attacked outside versus inside farms (coefficient: −1.1, *z* = 3.9, *P* < 0.001). Further, we found a significant effect of location ($\chi _1^2$ = 51.01, *P* < 0.001) and treatment ($\chi _2^2$ = 253.71, *P* < 0.001) on the total number of predators that attacked swarms (see Table S2 for model coefficients). Again, significantly more individual predators attacked the live mysid swarms compared to the empty bag control (coefficient: 4.7, *z* = 7.5, *P* < 0.001), there was no difference in the number of predator individuals that attacked the controls (coefficient: 0.4, *z* = 0.5, *P* = 0.601) and more individuals attacked outside versus inside farms (coefficient: −2.1, z = −6.8, *P* < 0.001, Fig. 2c). Our second predation experiment tested whether the persistence of naturally occurring swarms is dependent on the presence of damselfish. This two-part experiment compared the number of strikes on swarms when damselfish were present or experimentally removed compared to a baseline predation rate (Fig. S1). During the test period, we found that the number of strikes on swarms increased significantly when damselfish were removed (10.6 ± 4.378 SE; *V* = 0, *P* = 0.004) but not when they continued to defend their farms (0.4 ± 0.214 SE; *V* = 0, *P* = 0.089).

### Effect of mysids on damselfish behavior and condition

**Fig. 3:**
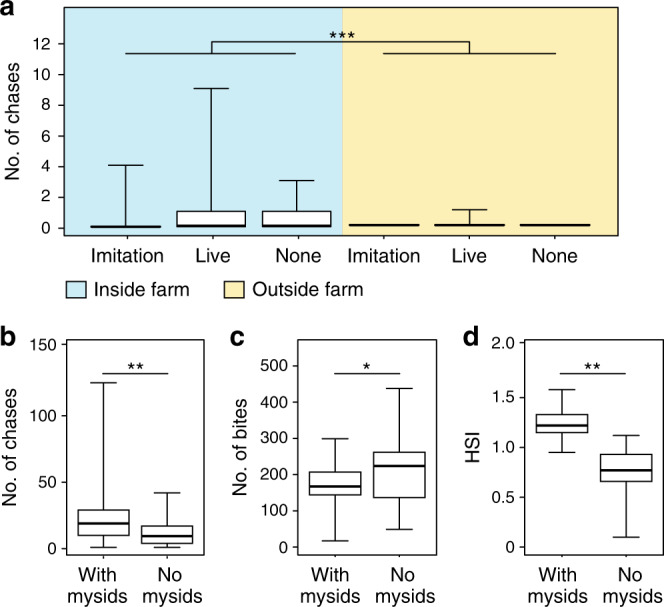
Behavior of longfin damselfish indicative of domesticator role. Number of chases by damselfish (*Stegastes diencaeus*) towards other fishes during (**a**) predation experiment (significant effect of location (zero-inflated GLMM): $\chi _1^2$ = 68.95, *P* < 0.001; significant effect of treatment (zero-inflated GLMM): $\chi _2^2$ = 6.07, *P* = 0.048, *n* = 30 damselfish farms with 30 trials inside the farm and 30 trials conducted outside of the farm) (post-hoc test included an mvt correction) and (**b**) 30-min behavioral observations (significant effect of farm type (glm): *F*_1, 57_ = 5.4, *P* = 0.024, *n* = 60 fish observed, including 30 that hosted mysids within their farm and 30 that did not host mysids within their farm). (**c**) Number of bites on farmed substrate by damselfish during 30-min behavioral observations (significant effect of farm type (glm): *F*_1, 58_ = 8.14, *P* = 0.006, *n* = 60 fish observed, including 30 that hosted mysids within their farm and 30 that did not host mysids within their farm) and (**d**) hepatosomatic index (HSI) of fish that either did or did not host mysids (significant effect of farm type (glm): *F*_1, 58_ = 82.2, *P* < 0.001, *n* = 60 fish, including 30 that hosted mysids within their farm and 30 that did not host mysids within their farm). Treatments for (**a**) are outlined in the Fig. 2 legend. Boxplots show median values (horizontal lines), interquartile range (boxes) and minimum and maximum values (whiskers). Asterisks indicate significant differences (*P* < 0.05 = *, *P* < 0.01 = **, *P* < 0.001 = ***). Note that while our analysis found a significant effect of treatment on the number of chases in the predation experiment (3a, *P* = 0.048), the more conservative multiple comparison post-hoc test found a slightly non-significant result (*P* = 0.089).

Our results indicate that the protection received by farm-associated mysids is directly related to damselfish behavior. Our first predation experiment revealed significant effects of location ($\chi _1^2$ = 68.95, *P* < 0.001) and treatment ($\chi _2^2$ = 6.07, *P* = 0.048) on the number of aggressive interactions between damselfish and predators, suggesting that hosting mysids comes at a cost (Fig. 3a). Specifically, we found that significantly more chases occurred inside versus outside farms (coefficient: 4.0, *z* = 4.0, *P* < 0.001), there was no difference in the number of chases between the two controls (coefficient: −0.1, *z* = −0.3, *P* = 0.797) and a marginally non-significant effect between the live mysid swarm compared to the empty bag control (coefficient: 0.7, *z* = 2.0, *P* = 0.051). Timed observations of farms with and without swarms provided further evidence of this cost: those hosting mysids engaged in significantly more defensive interactions (*F*_1, 57_ = 5.4, *P* = 0.024, Fig. 3b), expended effort to maintain frequent physical contact with swarms (interactions occurred 3.5 ± 0.6 SE. times per half hour observation and in 76.67% of behavioral observations, *n* = 30) and had significantly lower feeding rates (*F*_1, 58_ = 8.14, *P* = 0.006, Fig. 3c). However, despite these costs, mysid-hosting damselfish exhibited a significantly higher hepatosomatic index (*F*_1, 58_ = 82.2, *P* < 0.001), indicating greater energy storage and thus improved body condition (Fig. 3d). In addition to the lack of attraction to farmed turf odor, mysids also showed no preference between mysid-associated and non-mysid-associated longfin damselfish in an olfactory choice experiment (*t*_29_ = −1.2, *P* = 0.247), suggesting differences in body condition result from hosting mysids, as opposed to mysids associating with more robust damselfish. Despite small invertebrates forming an important supplementary component of farming damselfish diets^26^, longfin damselfish rarely attempted to feed on associated swarms, with only three suspected strikes seen in 15-h of observations. While damselfish with and without associated mysids exhibited behavioral differences, the estimated area of farms with mysids (2.598 m^2^ ± 0.383, *n* = 30) and without mysids (1.995 m^2^ ± 0.284, *n* = 30) was not significantly different (*W* = 562.2, *P* = 0.095).

### Effect of mysids on farm algae and nutrient availability

**Fig. 4:**
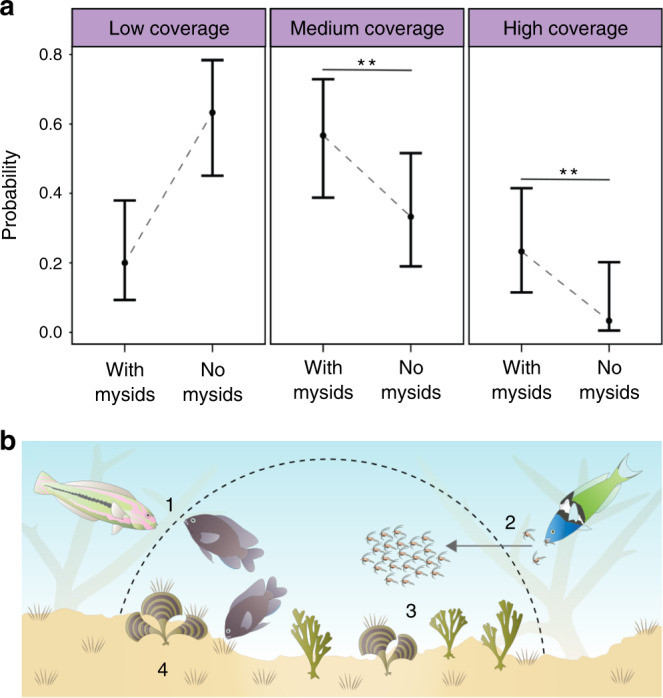
Effect of mysids on farmed algae and overview of damselfish-mysid relationship. (**a**) The impact of mysid presence on the predicted probabilities (model predicted probabilities ±95% confidence intervals) of low (<10%), medium (10–30%), and high (>30%) brown algae (Ochrophyta) coverage in longfin damselfish farms. Farms with associated mysids (*n* = 30) were more likely to have medium (*P* = 0.004) or high (*P* = 0.005) coverage than farms without associated mysids (*n* = 30) based on a multinomial logistic regression model (see Table S3). Asterisks indicate significant differences (*P* < 0.05 = *, *P* < 0.01 = **, *P* < 0.001 = ***). (**b**) The damselfish-mysid relationship, where (1) the niche created by the territorial, algae farming longfin damselfish provides (2) a protective refuge to mysids, leading to (3) increased survival. In turn, mysids provide (4) a predictable supply of nutrients, enriching the algae community and thus the quality of food available for damselfish.

To investigate why fish hosting mysids exhibited better body condition, we examined the composition of the underlying algae communities. Algal composition within mysid-associated farms was significantly different to those without mysids (Fig. 4). Mysid-associated farms contained a significantly higher proportion of Ochrophyta (brown algae; dominated by *Dictyota* and *Padina*) (multinomial logistic regression model; Table S3; medium Ochrophyta coverage: *P* = 0.004; high Ochrophyta coverage: *P* = 0.005). Fleshy macroalgae such as Ochrophyta increases the structural complexity of damselfish farms and can serve as substratum for the growth of palatable turf-algae, the preferred food source of territorial damselfish^34^. In addition, greater complexity would likely increase the abundance and diversity of algae-associated invertebrates^35^. As the presence of these algae are indicative of high nutrient availability^36^, we examined the potential enrichment effects that mysid swarms could have on associated farms. Swarm density in the field was conservatively estimated at 192 mysids L^−1^ ± 70 SE, with an average swarm size of 508 mysids ± 64 SE (*n* = 30 swarms collected). At this density, mysid swarms produce substantial quantities of biologically available nutrients, with captive swarms (at 200 mysids L^−1^) producing 0.58 mgL^−1^ ± 0.02 SE of NH_3_–N and 0.21 mgL^−1^ ±0.01 SE of *P* over an 8 h daylight period. Given the rapid rates of nutrient uptake by turf and macroalgae^37^, importance of these nutrients for algal growth^38^, distance from swarms to benthos, and limited water movement at the study site^39^, this daily nutrient supplement is consistent with mysids driving differences in algae composition, through the ‘fertilization’ of algae with their waste.

## Discussion

Our results indicate that the interactions between farming damselfish and farm-associated mysids comprise a domesticator-domesticate relationship. Damselfish provide significant protection to mysids that reside within farm boundaries, and mysids provide a consistent supply of fertilizer to the farm’s tended algae, which results in mysid-hosting damselfish exhibiting better body condition than those without mysids. Therefore, in this tripartite mutualism the domesticate (mysids) is supported due to an indirect resource it provides, which is distinct from many domesticator-domesticate relationships. However, this bares similarity to aspects of fungal agriculture in ants, with symbiotic fungal parasite-inhibiting bacteria maintained by the ants to protect their primary crops^40^. The presence of non-mysid associated longfin damselfish suggests that these fish create farms for purposes unrelated to mysid domestication. However, the lack of reef-associated mysids outside of farms at our study site suggests that these mysids have an obligate reliance on the niche created by the damselfish (i.e., farms) for survival within this predator-rich environment. These results are consistent with the hypothesized behavioral processes that underpin domestication via the commensal pathway^4,17^.

Both damselfish and mysids displayed behavioral traits indicative of a ‘domestication syndrome’^41,42^. For instance, mysids were consistently associated with the same damselfish farms, reacted positively to cues from their damselfish partner but either negatively or neutrally to cues from other species, and displayed low reactivity to damselfish. Similarly, while damselfish are territorial, exhibit aggression towards organisms that approach their farm and will consume small invertebrates, this aggression was not directed towards mysids. Docility towards mysids was observed, despite hosting swarms requiring a greater investment in territorial defense, suggesting that damselfish actively facilitate their presence. These behaviors are consistent with the hypothesized decrease in aggression that is essential to enable domestication^10^ and suggests these relationships have progressed beyond the opportunistic associations that can act as precursors to domestication and instead resemble a primitive domesticator-domesticate relationship. Further, while not the aim of this study, our behavioral evidence in support of domestication via the commensal pathway lays the foundation for future work to determine the temporal and spatial stability of these relationships, examine additional fitness metrics, and investigate evidence of genetic or morphological changes in both fish and mysids, which would signal further depth of these domesticatory partnerships. The relationships between humans and domestic animals are no doubt more complex than that described here; however, this study demonstrates that animal domestication by non-human vertebrates can occur, and provides experimental evidence that the opportunistic use of a novel niche by a proto-domesticate can lead to its subsequent domestication under natural conditions.

The facultative nature of the partnership between damselfish and mysids makes this system well-suited to disentangle the precursory behaviors associated with domestication. Evidence consistent with the commensal pathway exists in human-led domestication systems. For example, archeological analysis of cat remains suggests they were drawn to humans due to the abundance of rodents near grain stores^21^. However, given the obligate nature of most domesticator-domesticate relationships, explicitly demonstrating the initial factors associated with domestication has proven difficult^43^. Our experimental data, by contrast, clearly demonstrates that protection from predators is a key benefit conferred to mysids through their association with damselfish, with the persistence of swarms dependent on damselfish. Likewise, comparisons of damselfish HSI indicates that hosting mysids is associated with increased fish body condition. Together, these results provide strong support for the hypothesis that predation is selecting a domesticator-domesticate relationship between farming damselfish and mysids. Given that selection by predators can promote the widespread emergence^44^, convergence^45^ and breakdown^46^ of mutualisms, we suggest that predation-mediated domesticator-domesticate relationships, and the commensalisms that likely act as precursors, may be more common than is currently recognized. For example, species that can adapt to, and exist within, human-modified environments often experience reduced predation and increased fitness due to the inability of their predators to also exploit these habitats^47–49^. If humans began deriving a consistent resource from these types of species, the relationships may begin transitioning from a one-sided commensalism towards a proto-domesticator-domesticate relationship, and we suggest that this also may have happened in our prehistory and between other non-human organisms.

Our ability to domesticate other organisms has contributed to the success of our species and transformation of the world’s ecosystems^1,2^. The key contribution of this study is that we provide experimental evidence in support of the hypothesized commensal pathway to animal domestication. Unlike other proposed pathways, which rely on the coordinated manipulation of a proto-domesticate population (prey pathway) or conscious intention to domesticate another species (direct pathway), the ecological commonness of niche construction behaviors makes the commensal pathway an excellent candidate hypothesis for critically examining the ecology and process of domestication. We therefore emphasize the potential insights that can be made into domestication through the study of facultative relationships between non-human organisms.

## Methods

### Study location and species

Field research and sample collection was conducted on the shallow reef habitat surrounding the Smithsonian’s Carrie Bow Cay Research Station, Belize (16°48’9.8316”N, 88°4’54.8148”W) between January-April 2018. Mysid swarms, identified primarily as *Mysidium integrum*^50^, are present on these reefs year-round, and this species was used in all experiments. Six damselfish species in the genus *Stegastes* were present at the study site. Three of these, the longfin damselfish (*Stegastes diencaeus)*, the phylogenetically similar dusky damselfish (*Stegastes adustus)*^31^, and threespot damselfish (*Stegastes planifrons*), can be characterized as ‘intensive territorial grazers’ or ‘farmers’ that tend and aggressively defend the turf-algae communities on which they feed^24^. The others, the bicolor damselfish (*Stegastes partitus*), cocoa damselfish (*Stegastes variabilis*) and beaugregory (*Stegastes leucostictus*) tend turf-algae to a lesser degree and display limited territoriality. As the most common intensive-farming species, the longfin damselfish was used in all experiments.

### Analytical software

Analyses were conducted using R^51^. Generalized linear mixed models (GLMMs) were fitted with the *lme4* package^52^ and zero-inflated GLMMs were fitted with the *glmmTMB* package^53^. The multinomial logistic regression model used for algal analysis was performed with the packages *nnet*^54^ and *effects*^55^.

### Associations between mysids and damselfish farms

To determine whether mysid swarms were associated with *Stegastes* farms, we conducted a series of transects. Thirty 30-m transects were laid haphazardly across the study site. For 1 m on each side of the transect we recorded: the total number of swarms, the total number of *Stegastes* and whether a swarm was associated with a *Stegastes* farm. Each *Stegastes* was recorded to species. We used a $\chi$^2^ test to investigate whether the presence of intensive-farming *Stegastes* was associated with swarm presence.

### Mysid swarm movement and site fidelity

*Mysidium* swarms will often leave the substrate at dusk to feed in the water column, and isotope tagging indicates that swarms regroup at the same location each morning where they remain during daylight hours^29^. To test whether swarms at our study site followed this pattern, and whether swarms remained associated with the same *Stegastes* farms, we tested for site fidelity across a 20-day period. Thirty locations within longfin damselfish farms that had mysid swarms were tagged with numbered flagging tape. Thirty longfin damselfish farms where swarms were absent were also tagged. All locations were tagged between 9 and 10 a.m. Each location was visited at day 1, 10, and 20 post-tagging, 1-h post-sunrise and 1-h post-sunset, with swarm presence or absence recorded. In addition to formal rechecking, sites were frequently reassessed throughout this period, both during the day and at night, to confirm the consistency of the patterns recorded. We used a Friedman test to investigate whether swarms exhibited site fidelity to particular farms during the day over the 20-day period. The full and final model included time as a predictor and farm identification as a blocking factor.

### Mysid responses to habitat-related olfactory cues

Choice experiments were conducted to determine whether mysids used olfactory cues to actively seek out intensive-farming damselfish^33^. Experiments were conducted using a two-channel choice flume (13 cm length × 4 cm width)^56^. Header tanks contained two separate water sources, each gravity fed into separate sides of the flume at equivalent volumes (~100 mL min^−1^). The flume design ensures that once laminar flow is achieved each water mass remains separated on either side of the main chamber with no areas of turbulence or eddies, presenting an individual placed in the center with a choice between the two separate water sources/olfactory cues. Regular dye tests confirmed laminar flow and that the two water sources remained separated.

Five separate cue combinations were tested. Four cues were tested against a seawater control (seawater with no added cue): longfin damselfish (putative mutualism partner), farmed turf (putative mutualism partner’s environment), bicolor damselfish (non-intensive-farming damselfish that did not associate with mysids), and slippery dick wrasse (*Halichoeres bivittatus*, a diurnal predator of mysids). The fifth combination was a mysid-associated longfin damselfish versus a non-mysid-associated longfin damselfish. Cues were prepared by soaking an individual fish, or turf-covered rock from a longfin damselfish farm, in 10 L of seawater from the Carrie Bow Cay flow-through system for 1-h. All selected fishes and turf pieces had a similar biomass to minimize variation in cue concentration. For each trial set, both the cue and seawater control were produced concurrently, with both buckets sitting adjacent with constant aeration for the 1-h period. In this way, both cue and control had matching salinity, temperature, and O_2_ levels, minimizing any opportunity for behavioral bias due to the physical properties of the water and ensuring proper laminar flow. Each cue combination was split into three blocks: three separate fish or turf-covered rocks (one per block) were used to prepare treatments, with ten replicates obtained from each block (a total of *n* = 30 per combination). Individual mysids were only used in one trial.

All trials were conducted blind, with the tester having no knowledge of the cues tested or the side on which each cue was placed. A second observer was also present at all times. For each trial, a mysid was placed at the downstream center of the flume chamber. Following a 2-min habituation period, its position on either the left or right side of chamber was recorded at 5-s intervals for 2-min. Water sources were then switched to the opposite sides, and the chamber was allowed to flush for 1-min. The 2-min habituation period and 2-min test period were then repeated to exclude the possibility that mysids were exhibiting a side preference (i.e., spending 100% of time on one side of the chamber despite the water source being switched midway through the trial). Mysids that exhibited a side preference (*n* = 6 of 156) were excluded from analysis. Data were analyzed using paired *t*-tests, except for the longfin damselfish versus seawater comparison. Here, a Wilcoxon signed-rank test was used because these data did not meet the assumption of normality.

### Effect of predation on the damselfish-mysid relationship

To first test whether mysids receive protection by residing within the boundaries of longfin damselfish farms we conducted a predation-risk experiment. Sixty trials were conducted (*n* = 30 inside, and *n* = 30 immediately outside of farms), which each consisted of three treatments: (1) live mysids, (2) an ‘imitation’ mysid control, and (3) a seawater control. Each treatment consisted of a weighted 3.5 L polyethylene bag filled with seawater. The live mysid treatment consisted of 150 mysids. The ‘imitation’ mysid control was included to account for the presence of objects within the bag and consisted of 150 1 mm long sections of 4 mm diameter silicon tubing. These lightweight sections were slightly negatively buoyant and moved within the bag due to external water movement. Finally, the seawater control consisted of an empty seawater-filled bag. For each trial, bags were sequentially placed on the substrate in random order.

Each trial was 1-min in duration, during which the focal bag was filmed using an HD video camera (GoPro). After 1-min, this bag was removed and a 1-min rest period was observed. A bag containing the next treatment was then placed in the same location, and the 1-min trial was repeated. This same procedure was then repeated for the third bag. Videos were analyzed to compare: the number of fishes that attacked each bag, the number of strikes taken, the species of the attacker(s), and the number and species of fishes that came within 1 m of the bag but did not attack.

We used a zero-inflated GLMM with a Poisson distribution to test whether the number of strikes directed by predators at bags differed according to treatment (empty bag, artificial mysids, and live mysids), and location (inside versus outside of farm). The full and final model included treatment, location, and the interaction between treatment and location as fixed effects and trial as a random effect. We used a GLMM with a Poisson distribution to test whether the number of species that directed strikes at bags differed according to treatment and location. The full model included treatment, location and the interaction between treatment and location as fixed effects and trial as a random effect; however, the interaction was removed from the final model as it was found to be non-significant. We used a GLMM with a negative-binomial distribution to test whether the number of individuals that directed strikes at bags differed according to treatment and location. The full model included treatment, location and the interaction between treatment and location as fixed effects and trial as a random effect; however, the interaction was removed from the final model as it was found to be non-significant. Finally, we used a zero-inflated GLMM with a Poisson distribution to test whether the number of chases by longfin damselfish directed towards mysid predators differed according to treatment, location and the interaction between treatment and location. The full model included treatment, location and the interaction between treatment and location as fixed effects and trial as a random effect; however, the interaction was removed from the final model after being found to be non-significant.

In addition, to test whether the persistence of naturally occurring swarms was dependent on the protection damselfish provide, we conducted a second field-based predation experiment. Thirty trials were conducted (*n* = 15 treatment, and *n* = 15 control) with replicates for both conducted in a random order. For treatment trials, a swarm within a damselfish farm was observed on SCUBA from a distance of 2 m for a 5-min period. During this time, all strikes on the swarm by predatory fishes were recorded with this number taken as the baseline predation rate. Immediately following this period, a second 5-min observation was conducted during which a second diver actively prevented damselfish from defending their territory, pressuring fish into reef structure by gesturing at them using a fiberglass pole. During this second period, all strikes on the swarm were again recorded with this number taken as the change in predation rate. Control trials accounted for the effect of the second diver’s actions on predator behavior. Control period 1 was as above; however, during the second period, the second diver made movements and noise using the pole but did not direct this at damselfish, allowing them to continue to defend their farm. All strikes on the swarm were recorded. During control trials, damselfish did not react to the second diver’s actions indicating that their territorial behavior was not affected. Differences in strikes between periods were determined using Wilcoxon signed-rank tests.

### Effect of mysids on damselfish behavior

We conducted field observations to determine whether mysid-associated longfin damselfish behaved differently to those without mysids. Adult longfin damselfish that were (*n* = 30), or were not (*n* = 30), associated with swarms were observed for 30-min. For each observation, the focal fish was observed on SCUBA from a distance of at least 2-m. During each observation we recorded the number of bites on the farmed substrate, the number of strikes directed towards the swarms, the number of chases directed towards intruding fishes, the number of chases directed towards intruding fishes attempting to feed on farm-associated mysids and the number of non-aggressive interactions between the focal fish and the swarm. At the end of each observation, we recorded the total number of longfin damselfish associated with each farm with only one observation made per farm. An estimate of farm area was also made at this point by using a transect tape to measure the maximum length and width across the area that was actively defended and tended during the observation period.

We used a GLM with a Gaussian distribution to determine whether the number of chases by longfin damselfish was associated with the presence or absence of mysids. The full model included farm type (mysids present or absent), longfin damselfish group size, and the interaction between farm type and group size as fixed effects; however, the interaction between farm type and group size, and group size were removed from the final model as they were found to be non-significant. A GLM with a Gaussian distribution was used to test whether the number of bites on farmed substrate by focal longfin damselfish was associated with the presence or absence of mysids. The full model included farm type (mysids present or absent) longfin damselfish group size and the interaction between farm type and group size as fixed effects; however, the interaction between farm type and group size, and group size were removed from the final model because they were found to be non-significant. Whether farm area differed between farms with and without mysids was determined using a Wilcoxon rank sum test.

### Effect of mysid swarms on longfin damselfish body condition

To determine the effect of mysid presence on longfin damselfish body condition, we compared the hepatosomatic index (HSI) of damselfish with and without mysids in their farms. This measure can reflect the amount of stored energy in the liver, and thus it can indicate of the relationship between diet and physical condition in damselfishes^57–60^. Thirty adult longfin damselfish (75–100 mm TL) were sampled from farms with or without associated mysids. Damselfish were collected on snorkel using hand nets and a 1:3:7 clove oil/ethanol/seawater mixture. Prior to euthanasia, fish were maintained in a 20 L flow-through aquaria for 24-h. Fish were not fed during this period. Fish were euthanized by immersion in a clove oil/ethanol/seawater solution to induce anesthesia followed by immersion in an ice slurry. Once euthanized, fish were measured (SL and TL) and weighed. The liver of each fish was removed and weighed, and the alimentary canal checked to confirm that all digested matter was evacuated. The HSI of each fish was calculated as the proportion of total weight contributed by the liver [(liver weight (g)/total weight (g)) × 100]. We used a GLM with a Gaussian distribution to test the effect of mysid presence on hepatosomatic index (HSI). The full model included damselfish length, farm type (mysids present or absent) and the interaction between damselfish length and farm type as fixed effects; however, the interaction and damselfish length were removed from the final model as they were found to be non-significant.

### Effect of mysid swarms on algal composition within damselfish farms

Algal composition was assessed to determine the effect of mysid swarms on algae within longfin damselfish farms. Sixty farms were analyzed: 30 with and 30 without swarms. Three 20 cm × 20 cm quadrats were placed haphazardly within each farm, and a series of four photographs were taken, including one overhead shot encompassing the entire quadrat and three macro shots of the algae within the quadrat. Within each quadrat, algal composition and coverage was determined to phylum, including Chlorophyta, Rhodophyta, and Ochrophyta. Percent cover of these groups was assigned based on a categorical classification scheme: low (<10% coverage); medium (10–30% coverage); and high (>30% coverage). To obtain a single assessment of algal composition for each phylum within each farm, the categorical classification was averaged across the three quadrats photographed in each farm. We used a multinomial logistic regression model to assess how the percent cover of Ochrophyta within farms was affected by swarm presence. The response within each model was multinomial: low, medium, or high percent coverage of Ochrophyta. The model included the fixed effect of mysid presence or absence.

### Estimates of mysid swarm density

Surveys were conducted to determine the average size and density of farm-associated swarms. The size and area of 30 focal swarms were determined by measuring the length, width and height across the widest points when first observed. Each swarm was then collected using hand nets and returned to the laboratory where the total number of mysids within each swarm was counted. Finally, swarm density was estimated by calculating the maximum ellipsoid volume based on the measured axes and dividing this volume by the total number of mysids, giving an estimate of mysids mL^−1^.

### Mysid waste excretion and nutrient availability

We used an aquarium experiment to determine if mysid swarms produce key nutrients at concentrations that could enhance benthic algal growth. Artificial seawater was produced at sunrise by mixing deionized fresh water with a phosphate and nitrogen-free aquarium salt (Instant Ocean^®^ Sea Salt) to 35ppt salinity. Once mixed, the absence of phosphate and ammonia was confirmed through color comparison using laboratory-grade test kits (Hach PO-19A test kit, Hach NI-SA test kit), and the temperature and pH of the water were also measured. Seawater was then distributed into a series of 1 L plastic containers, along with one air stone per container. Containers were covered to prevent water loss through evaporation.

Mysids were collected using hand nets and returned to the lab where they were allowed to habituate for 30-min in a bucket containing 3 L of artificial seawater. For sorting, mysids were removed from the habituation bucket using a hand net and placed into a petri dish containing artificial seawater. Mysids were individually selected using a sterile plastic pipette and moved into a second petri dish containing artificial seawater. The water in the second petri dish was then removed using the pipette, and mysids were placed into the appropriate container corresponding to one of three treatments: 0 mysids L^−1^ (*n* = 30), which served as a control, 100 mysids L^−1^ (*n* = 30) and 200 mysids L^−1^ (*n* = 30). These densities were selected as they are representative of the range found during the swarm density surveys. Each day, containers were haphazardly assigned a treatment prior to sorting, with an equal number of replicates for each treatment (*n* = 5) conducted each day. Trials were conducted from 08:00 to 16:00. This period was selected to represent the daylight hours when mysids are present, but was also short enough to prevent stress due to nutrient accumulation. Following this 8-h period, mysids were removed, and the concentration of phosphorous (P) and nitrogen-ammonia (NH_3_–N) in each container were recorded to the nearest 0.1 mgL^−1^ through color comparison. The temperature, salinity, and pH of each container were also recorded at this point.

### Reporting summary

Further information on research design is available in the Nature Research Reporting Summary linked to this article.

## Supplementary information

Supplementary Information

Reporting Summary

## Data Availability

All data supporting the findings of this study are available at: 10.5281/zenodo.4210945. Raw video and photograph files are available from the corresponding author upon reasonable request. The source data underlying Figs. 1–4 and Supplementary Fig. 1 are provided as the Source Data file. Source data are provided with this paper.

## Source data

Source Data

## Code availability

All code required to replicate the analyses in this study is available at: 10.5281/zenodo.4210945.

## References

[1] Kareiva P, Watts S, McDonald R, Boucher T (2007). Domesticated nature: shaping landscapes and ecosystems for human welfare. Science.

[2] Boivin NL (2016). Ecological consequences of human niche construction: examining long-term anthropogenic shaping of global species distributions. Proc. Natl Acad. Sci. USA.

[3] Mueller UG, Gerardo NM, Aanen DK, Six DL, Schultz TR (2005). The evolution of agriculture in insects. Annu. Rev. Ecol. Evol. Syst..

[4] Larson G, Fuller DQ (2014). The evolution of animal domestication. Annu. Rev. Ecol. Evol. Syst..

[5] Zeder MA (2015). Core questions in domestication research. Proc. Natl Acad. Sci. USA.

[6] Frantz LAF, Bradley DG, Larson G, Orlando L (2020). Animal domestication in the era of ancient genomics. Nat. Rev. Gen..

[7] Darwin CR (1868). The Variation of Animals and Plants under Domestication.

[8] Galton F (1883). Inquiries into Human Faculty.

[9] Trut LN (1999). Early canid domestication: the farm-fox experiment. Am. Sci..

[10] Trut L, Oskina I, Kharlamova A (2009). Animal evolution during domestication: the domesticated fox as a model. BioEssays.

[11] Farrell BD (2001). The evolution of agriculture in beetles (Curculionidae: Scolytinae and Platypodinae). Evolution.

[12] Aanen DK (2002). The evolution of fungus-growing termites and their mutualistic fungal symbionts. Proc. Natl Acad. Sci. USA.

[13] Schultz TR, Brady SG (2008). Major evolutionary transitions in ant agriculture. Proc. Natl Acad. Sci. USA.

[14] Ivens ABF, Kronauer DJC, Pen I, Weissing FJ, Boomsma JJ (2012). Ants farm subterranean aphids mostly in single clone groups - an example of prudent husbandry for carbohydrates and proteins?. BMC Evol. Biol..

[15] Brooker RM, Feeney WE (2019). Animal domesticators. Curr. Biol..

[16] Vigne J-D (2011). The origins of animal domestication and husbandry: a major change in the history of humanity and the biosphere. C. R. Biol..

[17] Zeder MA (2012). The domestication of animals. J. Anthropol. Res..

[18] Koster K (2009). Hunting dogs in the lowland neotropics. J. Anthropol. Res..

[19] Nygaard S (2016). Reciprocal genomic evolution in the ant–fungus agricultural symbiosis. Nat. Commun..

[20] Branstetter MG (2017). Dry habitats were crucibles of domestication in the evolution of agriculture in ants. Proc. R. Soc. B Biol. Sci..

[21] Hu Y (2014). Earliest evidence for commensal processes of cat domestication. Proc. Natl Acad. Sci. USA.

[22] Hata, H. & Ceccarelli, D. M. Farming behaviour of territorial damselfishes. In *Biology of Damselfishes* (eds. Frédérich, B. & Parmentier, E.) 140–170 (CRC Press, Boca Raton, 2016).

[23] Hata H, Kato M (2006). A novel obligate cultivation mutualism between damselfish and *Polysiphonia* algae. Biol. Lett..

[24] Emslie MJ (2012). Regional-scale variation in the distribution and abundance of farming damselfishes on Australia’s Great Barrier Reef. Mar. Biol..

[25] Mauchline J (1980). The biology of mysids. Adv. Mar. Biol..

[26] Dromard CR (2013). Resource use of two damselfishes, *Stegastes planifrons* and *Stegastes adustus*, on Guadeloupean reefs (Lesser Antilles): inference from stomach content and stable isotope analysis. J. Exp. Mar. Biol. Ecol..

[27] Emery AR (1968). Preliminary observations of coral reef plankton. Limnol. Oceanogr..

[28] Hahn P, Itzkowitz M (1986). Site preference and homing behavior in the mysid shrimp *Mysidium gracile* (Dana). Crustaceana.

[29] Twining BS, Gilbert JJ, Fisher NS (2000). Evidence of homing behavior in the coral reef mysid *Mysidium gracile*. Limnol. Oceanogr..

[30] Allgeier JE, Burkepile DE, Layman CA (2017). Animal pee in the sea: consumer-mediated nutrient dynamics in the world’s changing oceans. Glob. Chang. Biol..

[31] Mullen SP, Little K, Draud M, Brozek J, Itzkowitz M (2012). Hybridization among Caribbean damselfish species correlates with habitat degradation. J. Exp. Mar. Biol. Ecol..

[32] Modlin RF (1993). Population parameters, life cycle, and feeding of *Mysidium columbiae* (Zimmer) in the waters surrounding a Belizian mangrove cay. Mar. Ecol..

[33] Jutfelt F, Sundin J, Raby GD, Krång A‐S, Clark TD (2017). Two-current choice flumes for testing avoidance and preference in aquatic animals. Methods Ecol. Evol..

[34] Ceccarelli DM, Jones GP, McCook LJ (2005). Effects of territorial damselfish on an algal-dominated coastal coral reef. Coral Reefs.

[35] Kramer MJ, Bellwood DR, Bellwood O (2012). Cryptofauna of the epilithic algal matrix on an inshore coral reef, Great Barrier Reef. Coral Reefs.

[36] Thacker R, Ginsburg D, Paul V (2001). Effects of herbivore exclusion and nutrient enrichment on coral reef macroalgae and cyanobacteria. Coral Reefs.

[37] den Haan J (2016). Nitrogen and phosphorus uptake rates of different species from a coral reef community after a nutrient pulse. Sci. Rep..

[38] Larned ST (1998). Nitrogen- versus phosphorus-limited growth and sources of nutrients for coral reef macroalgae. Mar. Biol..

[39] Kjerfve B, Rützler K, Kierspe GH (1982). Tides at Carrie Bow Cay, Belize. Smithsonian. Contrib. Mar. Sci..

[40] Currie CR, Poulsen M, Mendenhall J, Boomsma JJ, Billen J (2006). Coevolved crypts and exocrine glands support mutualistic bacteria in fungus-growing ants. Science.

[41] Wilkins AS, Wrangham RW, Tecumseh Fitch W (2014). The “domestication syndrome” in mammals: a unified explanation based on neural crest cell behavior and genetics. Genetics.

[42] Milla R, Osborne CP, Turcotte MM, Violle C (2015). Plant domestication through an ecological lens. Trends Ecol. Evol..

[43] Frantz LAF, Bradley DG, Larson G, Orlando L (2020). Animal domestication in the era of ancient genetics. Nat. Rev. Gen..

[44] Canestrari D (2014). From parasitism to mutualism: unexpected interactions between a cuckoo and its host. Science.

[45] Feeney WE (2019). Predation drives recurrent convergence of an interspecies mutualism. Ecol. Lett..

[46] Palmer TM (2008). Breakdown of an ant-plant mutualism follows the loss of large herbivores from an African savanna. Science.

[47] Hebblewhite M (2005). Human activity mediates a trophic cascade caused by wolves. Ecology.

[48] Gering JC, Blair RB (2006). Predation on artificial bird nests along an urban gradient: predatory risk or relaxation in urban environments?. Ecography.

[49] Geffroy B (2020). Evolutionary dynamics in the Anthropocene: life history and intensity of human contact shape antipredator responses. PLoS Biol..

[50] Wittmann KJ, Wirtz P (2019). Revision of the amphiamerican genus *Mysidium* Dana, 1852 (Crustacea: Mysida: Mysidae), with descriptions of two new species and the establishment of two new subgenera. Eur. J. Taxon..

[51] R Core Team. (2019). R: a language and environment for statistical computing. R Foundation for Statistical Computing, Vienna, Austria. http://www.R-project.org

[52] Pinheiro J, Bates D, DebRoy S, Sarkar D (2018). nlme: linear and nonlinear mixed effects models. R. package version.

[53] Brooks ME (2017). glmmTMB balances speed and flexibility among packages for zero-inflated generalized linear mixed modeling. R J..

[54] Venables, W. N. & Ripley, B. D. *Modern Applied Statistics with S*. (Springer, New York, 2002).

[55] Fox J, Hong J (2009). Effect displays in R for multinomial and proportional-odds logit models: extensions to the effects package. J. Stat. Softw..

[56] Gerlach G, Atema J, Kingsford MJ, Black KP, Miller-Sims V (2007). Smelling home can prevent dispersal of reef fish larvae. Proc. Natl Acad. Sci. USA.

[57] McCormick MI (2003). Consumption of coral propagules after mass spawning enhances larval quality of damselfish through maternal effects. Oecologia.

[58] Gagliano M, McCormick MI (2004). Feeding history influences otolith shape in tropical fish. Mar. Ecol. Prog. Ser..

[59] Bapary MAJ, Amin MN, Takemura A (2012). Food availability as a possible determinant to initiation and termination of reproductive activity in the tropical damselfish *Chrysiptera cyanea*. Mar. Biol. Res..

[60] Rizky D, Mahardini A, Byun J, Takemura A (2020). Molecular cloning of insulin-like growth factor 3 (igf3) and its expression in the tissues of a female damselfish, *Chrysiptera cyanea*, in relation to seasonal and food-manipulated reproduction. Gen. Comp. Endocrinol..

