# Supplementary material for: Domestication via the commensal pathway in a fish-invertebrate mutualism
Source: Nat Commun. 2020 Dec 7;11:6253. doi: 10.1038/s41467-020-19958-5 (PMC7721709; doi:10.1038/s41467-020-19958-5)
Supplement: Supplementary file 1 — Supplementary Information [file 41467_2020_19958_MOESM1_ESM.pdf]

Supplementary Information for

Domestication via the commensal pathway in a fish-invertebrate mutualism

Authors:

Rohan M. Brooker*†, Jordan M. Casey, Zara-Louise Cowan, Tiffany L. Sih, Danielle L. Dixson, Andrea Manica, William E. Feeney†

*Correspond with: rohan.m.brooker@gmail.com

†Indicates equal contribution

This file includes:

Tables S1-S3
Fig S1

Table S1. Zero-inflated generalized linear mixed model outputs when testing how location (inside or outside of a longfin damselfish farm) and treatment (150 'imitation' mysid shrimps, 150 live mysid shrimps, or an empty seawater control) affects the number of strikes by predatory fishes on plastic bags during a field-based predation experiment. Significant values (P < 0.05) are in bold.

	**Estimate**	**Std. Error**	**z value**	**Pr(>|z|)**
(Intercept)	2.055	0.394	5.215	**<0.001**
HabitatST	-2.245	0.716	-3.137	**0.002**
TreatmentFM	-2.673	0.549	-4.866	**<0.001**
TreatmentM	2.284	0.353	6.475	**<0.001**
HabitatST:TreatmentFM	2.431	0.952	2.553	**0.011**
HabitatST:TreatmentM	0.894	0.717	1.247	0.212

Table S2. Generalized linear mixed model outputs when testing how location (inside or outside of a longfin damselfish farm) and treatment (150 'imitation' mysid shrimps, 150 live mysid shrimps, or an empty seawater control) affects the number of individual predators that made strikes on plastic bags during a field-based predation experiment. Significant values (*P* < 0.05) are in bold.

	**Estimate**	**Std. Error**	**z value**	**Pr(>|z|)**
(Intercept)	-3.8542	1.0047	-3.836	**<0.001**
HabitatST	0.6931	1.2053	0.575	0.565
TreatmentFM	1.3862	1.1003	1.26	0.208
TreatmentM	5.6801	0.9858	5.762	**<0.001**
HabitatST:TreatmentFM	-2.0794	1.6319	-1.274	0.203
HabitatST:TreatmentM	-2.5232	1.2151	-2.076	**0.038**

Table S3. Results from a multinomial logistic regression model that demonstrates the impact of mysid presence or absence on brown algae (Ochrophyta) percent coverage (low: <10% coverage; medium: 10-30% coverage; high: >30% coverage) inside longfin damselfish (*Stegastes diencaeus*) farms. *P*-values indicate that the treatment differs from the control as designated by the null hypothesis. Significant values ($p < 0.05$) are in bold.

**Ochrophyta**	**Variable**	**Coefficients**	**Std. Error**	**p-value**
Medium	Control	-0.642	0.391	--
	Mysids	1.683	0.615	**0.004**
High	Control	-2.944	1.026	--
	Mysids	3.098	1.167	**0.005**

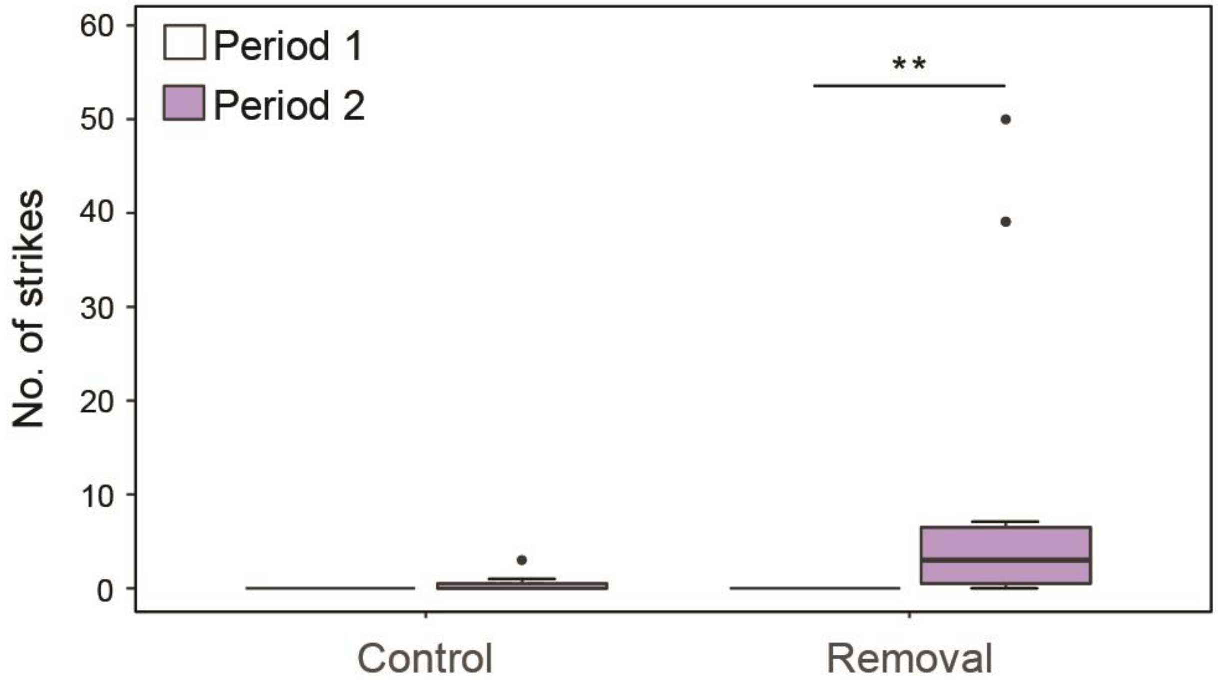

Fig. S1: Results of predation experiment testing if persistence of naturally occurring swarms is dependent on the presence of damselfish.

Change in the number of strikes by predatory fishes on naturally occurring mysid swarms when farming damselfish (*Stegastes diencaeus*) were either present (control n = 15) or absent (removal n = 15). In each case, period 1 represented the baseline predation rate over 5-min with damselfish present. During control period 2 (5-min), damselfish were present alongside a diver. During removal period 2 (5-min), damselfish were absent alongside a diver. The number of strikes on swarms increased significantly in period 2 when damselfish were absent (Wilcoxon signed-rank test: V=0, P=0.004) but not when they were present (Wilcoxon signed-rank test: V=0, P=0.089). Asterisks show significant differences ($P < 0.05$ = *, $P < 0.01$ = **, $P < 0.001$ = ***). Boxplots show median values (horizontal lines), interquartile range (boxes), and minimum and maximum values (whiskers). Outliers indicate values outside 1.5 times the interquartile range.
