# Supplementary material for: Domestication via the commensal pathway in a fish-invertebrate mutualism
Source: Nat Commun. 2020 Dec 7;11:6253. doi: 10.1038/s41467-020-19958-5 (PMC7721709; doi:10.1038/s41467-020-19958-5)
Supplement: Supplementary file 2 — Reporting Summary [file 41467_2020_19958_MOESM2_ESM.pdf]

nature research

Corresponding author(s): Rohan Brooker

Last updated by author(s): Nov 3, 2020

Reporting Summary

Nature Research wishes to improve the reproducibility of the work that we publish. This form provides structure for consistency and transparency in reporting. For further information on Nature Research policies, seeAuthors & Referees and theEditorial Policy Checklist .

Statistics

For all statistical analyses, confirm that the following items are present in the figure legend, table legend, main text, or Methods section.

n/a	Confirmed	
☐	☒	The exact sample size (*n*) for each experimental group/condition, given as a discrete number and unit of measurement
☐	☒	A statement on whether measurements were taken from distinct samples or whether the same sample was measured repeatedly
☐	☒	The statistical test(s) used AND whether they are one- or two-sided<br>*Only common tests should be described solely by name; describe more complex techniques in the Methods section.*
☐	☒	A description of all covariates tested
☐	☒	A description of any assumptions or corrections, such as tests of normality and adjustment for multiple comparisons
☐	☒	A full description of the statistical parameters including central tendency (e.g. means) or other basic estimates (e.g. regression coefficient) AND variation (e.g. standard deviation) or associated estimates of uncertainty (e.g. confidence intervals)
☐	☒	For null hypothesis testing, the test statistic (e.g. *F*, *t*, *r*) with confidence intervals, effect sizes, degrees of freedom and *P* value noted<br>*Give P values as exact values whenever suitable.*
☒	☐	For Bayesian analysis, information on the choice of priors and Markov chain Monte Carlo settings
☐	☒	For hierarchical and complex designs, identification of the appropriate level for tests and full reporting of outcomes
☐	☒	Estimates of effect sizes (e.g. Cohen's *d*, Pearson's *r*), indicating how they were calculated

Our web collection on statistics for biologists contains articles on many of the points above.

Software and code

Policy information about availability of computer code

Data collection	No software was used for data collection.
Data analysis	R coding for statistical analysis, version 3.6.3. Associated packages: effects, glmmTMB, lme4, nnet.

For manuscripts utilizing custom algorithms or software that are central to the research but not yet described in published literature, software must be made available to editors/reviewers. We strongly encourage code deposition in a community repository (e.g. GitHub). See the Nature Research guidelines for submitting code & software for further information.

Data

Policy information about availability of data

All manuscripts must include a data availability statement. This statement should provide the following information, where applicable:

- Accession codes, unique identifiers, or web links for publicly available datasets
- A list of figures that have associated raw data
- A description of any restrictions on data availability

All data supporting the findings of this study are available at: 10.5281/zenodo.4210945. Raw video and photograph files are available from the corresponding author upon reasonable request. The source data underlying Figs 1-4 and Supplementary Fig 1 are provided with the manuscript as the Source Data file.

Field-specific reporting

Please select the one below that is the best fit for your research. If you are not sure, read the appropriate sections before making your selection.

☐ Life sciences ☐ Behavioural & social sciences ☒ Ecological, evolutionary & environmental sciences

Ecological, evolutionary & environmental sciences study design

All studies must disclose on these points even when the disclosure is negative.

Study description	The aim of this study was to assess whether the apparent relationship between reef-associated damselfish and mysid shrimps constitutes a domesticator-domesticate relationship, and if this can be explained via the hypothesised commensal pathway. We achieved this by examining the ecology of both fish and mysids through a combination of field observations, manipulative experiments, and sample analysis. Pilot studies were conducted for the experiments, which were used to inform power analyses and determine sample sizes.
Research sample	This study used the interactions between longfin damselfish (Stegastes diencaeus) and a common species of mysid shrimp (Mysidum integrum) as its model. Both species are found associated with shallow reef communities throughout the Western Atlantic. Adult S. diencaeus of undetermined sex were used for all field- and laboratory-based components, with both fish and mysids collected from the shallow reefs surrounding Carrie Bow Cay Research Station for laboratory-based data collection. In addition, a predatory fish (Halichoeres bivittatus) and Stegastes sp. not associated with mysids (S. partitus) were collected for use in olfactory choice trials. The naturally occurring fish community was used to assess the effect of predation risk on damselfish-mysid interactions during field-based manipulative experiments and observations. To assess the effect of mysid presence on the underlying algal turf community, algal turf composition was compared between damselfish territories with and without mysids. Pilot studies were conducted for the experiments, which were used to inform sample choice.
Sampling strategy	Sampling comprised field-based transects, behavioural observations, specimen collections, field experiments, and aquarium experiments. Sample sizes we determined based off a combination of pilot studies in the field and estimates based off recent field- and aquarium-based behavioural research.
Data collection	In-water data collection (transects and distribution study, predation experiment, behavioural observations) were conducted by R Brooker, T Sih, and W Feeney. Olfactory choice trial data was collected by Z-L Cowan with assistance from R Brooker and W Feeney. Algal composition data was collected by J Casey using photographs collated by R Brooker. Longfin damselfish dissections were conducted by R Brooker with assistance from T Sih. Mysid excretion data was collected by R Brooker with assistance from Z-L Cowan.
Timing and spatial scale	Data included in this study were collected over a six-week period across two field trips in early 2018. Trip one ran from the 17th January to 14th February 2018 and trip two ran from the 11th to 25th April 2018. This time was sufficient to complete the experiments required within budget. All data were collected at Carrie Bow Cay Research Station and associated reef, Belize. This location was selected due to the presence of the study system and laboratory infrastructure.
Data exclusions	No data were excluded.
Reproducibility	Pilot studies were conducted prior to data collection to ensure consistency of methods used. A full description of the methodologies used is provided in the Methods and the data and full code necessary to reproduce the findings are provided through the Zenodo Data Repository links in the data and code accessibility statements. Photographs used for algal analysis and video recordings of predation experiment trials are available from the authors on reasonable request.
Randomization	Where applicable, the S. diencaeus and associated territories used were divided into two groups, those with and without associated mysids. Within each group, all replicates were then haphazardly chosen. For components where separation into these groups was not required, all replicates were haphazardly chosen based on first encounter.
Blinding	Blinding was used during olfactory choice trials to account for and remove observer bias. All trials were conducted blind, with the tester (Z-L Cowan) having no knowledge of the cue being tested or the side that each cue was placed (R Brooker and W Feeney ensured that Z-L Cowan was unaware of what was being tested until after all fluming data was collected). While blinding was not possible for other components because experiments were conducted in-field, experiments were video recorded and all field components were conducted by multiple individuals (R Brooker, T Sih and W Feeney). Photographs were also taken of farms for subsequent analysis, with this media (photos and videos) available from the authors on reasonable request.

Did the study involve field work? ☒ Yes ☐ No

Field work, collection and transport

Field conditions	Field (sea) conditions were generally calm. Weather was fair with intermittent rainfall.
Location	This study was conducted Carrie Bow Cay Research Station, South Water Caye Marine Reserve, Belize (16° 48' 9397" N, 88° 4' 54.912" W).
Access and import/export	Permission to conduct this study was granted under Marine Science Research Permit 0009-18 valid until 3/12/2018, issued by the Belize Fisheries Department, Ministry of Agriculture, Fisheries, Forestry, the Environment & Sustainable Development.
Disturbance	Only common plants and animals were used in manipulative experiments and collections. Disturbance to other wildlife was minimised by targeted collection of experimental animals. All other data collection was observational.

Reporting for specific materials, systems and methods

We require information from authors about some types of materials, experimental systems and methods used in many studies. Here, indicate whether each material, system or method listed is relevant to your study. If you are not sure if a list item applies to your research, read the appropriate section before selecting a response.

Materials & experimental systems

n/a	Involved in the study
☒	☐ Antibodies
☒	☐ Eukaryotic cell lines
☒	☐ Palaeontology
☐	☒ Animals and other organisms
☒	☐ Human research participants
☒	☐ Clinical data

Methods

n/a	Involved in the study
☒	☐ ChIP-seq
☒	☐ Flow cytometry
☒	☐ MRI-based neuroimaging

Animals and other organisms

Policy information about studies involving animals; ARRIVE guidelines recommended for reporting animal research

Laboratory animals	This study did not involve laboratory animals.
Wild animals	This study involved the use of wild fishes for field-based observational studies and manipulative experiments, as well as in laboratory-based manipulative experiments and body condition analysis. Transects involved recording the location of all fishes from the genus Stegastes (S. diencaeus, S. adustus, S. partitus, S. variabilis, S. leucostictus, S. planifrons). The predation experiment involved 30 focal S. diencaeus and miscellaneous wild predatory fishes. Behavioural observations involved 60 focal S. diencaeus and miscellaneous wild fishes. Olfactory choice trials involved 9 S. diencaeus, 3 S. partitus, and 3 Halichoeres bivittatus. Fishes were collected on snorkel using hand nets, and a 1:3:7 clove oil/ethanol/seawater mixture if needed and transported to the laboratory via bucket. All fishes used in this experiment were subsequently released following the completion of associated trials, as close to the capture location as possible. Body condition analysis involved the collection and euthanasia of 60 S. diencaeus. Fish were euthanized by immersion in a clove oil/ethanol/seawater solution to induce anesthesia followed by immersion in an ice slurry. All mysids used in the study were collected using hand nets and transported to the laboratory in 3.5L polyethylene bags. Following completion of experiments they were transported back to the reef in these bags.
Field-collected samples	Photographic sampling was used for algal analysis subsequent to field-based work. Fishes, mysids, and algae collected for experiments were held at the Carrie Bow Cay Research Station wet laboratory, which is fitted with a flow through sea water system. Animals and and algae were held in 20L flow-through aquaria containing ambient-temperature water drawn from a pipeline extending into the ocean. Each aquaria was fitted with an airstone to provide additional aeration. The wet laboratory receives natural light and so all animals were subject to a natural photoperiod while in captivity. All fishes and mysids collected for use in laboratory behavioural experiments were subsequently released following the completion of associated trials, as close to the capture location as possible. Fish collected for body condition analysis were maintained in flow-through aquaria for 24-hr prior to euthanasia and were not fed during this period.
Ethics oversight	Animal ethics for observations, manipulative experiments and collections involving fishes was issued by University of Delaware Institutional Animal Care and Use Committee (IACUC; approval number AUP 1300). Ethics approval was not required to use mysids in research.

Note that full information on the approval of the study protocol must also be provided in the manuscript.
